# Supplementary material for: Splice-Junction-Based Mapping of Alternative Isoforms in the Human Proteome
Source: Cell Rep. Author manuscript; Available in PMC 2020 Jan 15. (PMC6961840; doi:10.1016/j.celrep.2019.11.026)
Supplement: 2 [file NIHMS1546469-supplement-2.zip › DF1/PXD009737/TestisTrypsin/TestisTrypsin_8_KIAA1210_SLTATQVEPKEEEPNLPLVSEEEK.pdf]

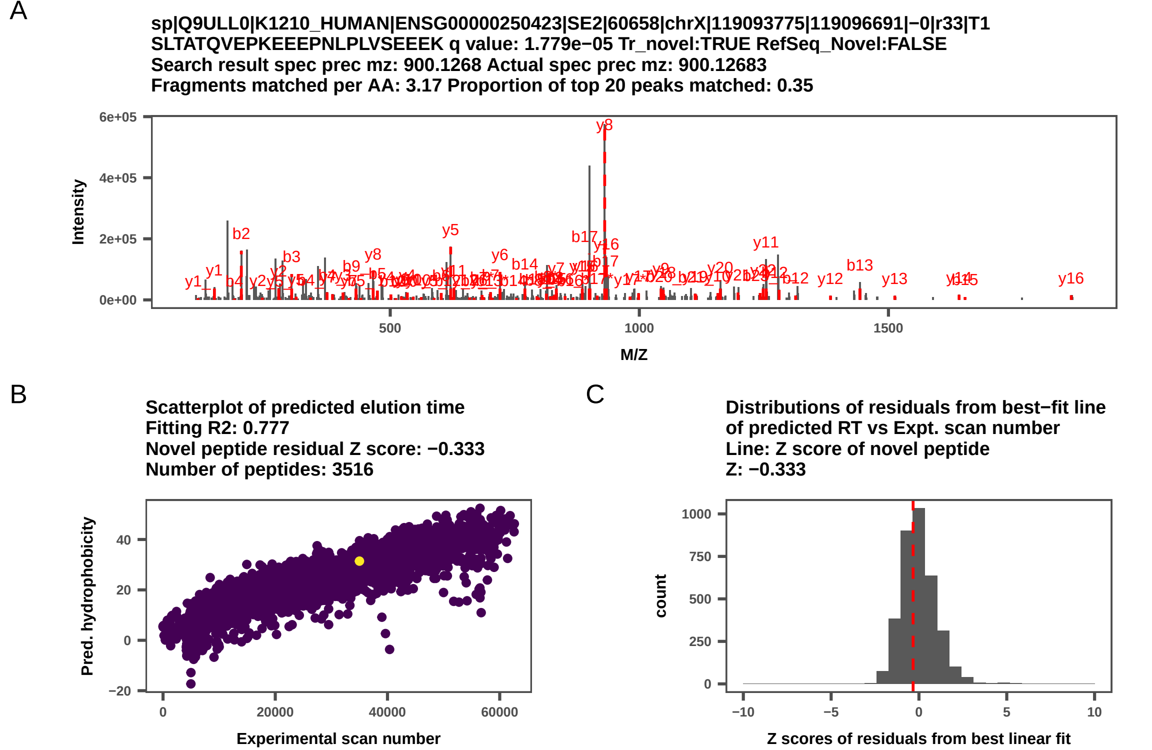
A
sp|Q9ULL0|K1210_HUMAN|ENSG00000250423|SE2|60658|chrX|119093775|119096691|−0|r33|T1
SLTATQVEPKEEEPNLPLVSEEEK q value: 1.779e−05 Tr_novel:TRUE RefSeq_Novel:FALSE
Search result spec prec mz: 900.1268 Actual spec prec mz: 900.12683
Fragments matched per AA: 3.17 Proportion of top 20 peaks matched: 0.35
Intensity
6e+05
4e+05
2e+05
0e+00
y8
b2
y5
b17
y16
y11
b3
y8
y6
b14
b13
y1
y2
b9
y12
y13
y16
500
1000
1500
M/Z
B
Scatterplot of predicted elution time
Fitting R2: 0.777
Novel peptide residual Z score: −0.333
Number of peptides: 3516
Pred. hydrophobicity
40
20
0
−20
0
20000
40000
60000
Experimental scan number
C
Distributions of residuals from best−fit line
of predicted RT vs Expt. scan number
Line: Z score of novel peptide
Z: −0.333
count
1000
750
500
250
0
−10
−5
0
5
10
Z scores of residuals from best linear fit
